# Infections in Children with Acute Lymphoblastic Leukemia

**DOI:** 10.3390/medicina60091395

**Published:** 2024-08-26

**Authors:** Silvije Šegulja, Klara Vranešević, Ana Đorđević, Jelena Roganović

**Affiliations:** 1 Department of Clinical Medical Studies, Faculty of Health Studies, University of Rijeka, 51000 Rijeka, Croatia; silvije.segulja@gmail.com; 2 Emergency Medicine Institute Osijek-Baranja County, 31000 Osijek, Croatia; klara.vranesevic@gmail.com; 3 Jadran-Galenski Laboratorij d.d., Svilno 20, 51000 Rijeka, Croatia; ana_dordevic@hotmail.com; 4 Children’s Hospital Zagreb, Klaiceva 16, 10000 Zagreb, Croatia; 5 Faculty of Biotechnology and Drug Development, University of Rijeka, Radmile Matejcic 2, 51000 Rijeka, Croatia

**Keywords:** acute lymphoblastic leukemia, child, infection, neutropenia

## Abstract

*Background and Objectives:* Infections are the most common and potentially life-threatening complications of the treatment of children with acute lymphoblastic leukemia (ALL). The aim of this study was to determine epidemiological, clinical, and microbiological characteristics of infections in pediatric patients with ALL. *Materials and Methods:* Twenty-three children (16 males and 7 females, with a mean age of 5.9 years (range of 1.3 to 12.2 years)) with ALL, treated at the Division of Hematology, Oncology, and Clinical Genetics, Department of Pediatrics, Clinical Hospital Center Rijeka, Croatia, from 1 January 2015 to 31 December 2020, were included in the study. *Results:* One hundred and four infectious episodes (IEs) were reported (an average of 4.5 IE per patient). IEs were more frequent in the intensive phases of antileukemic treatment. Neutropenia was present in 48 IEs (46.2%) with a duration greater than 7 days in 28 IEs (58.3%). The respiratory tract was the most common infection site (48.1%). We documented 49 bacterial (47.1%), 4 viral (3.9%), 4 fungal (3.9%), and 10 mixed isolates (9.6%), while in 37 IEs (35.6%), a pathogen was not isolated. The most common causes of bacteremia were coagulase-positive staphylococci. The most frequent empirical therapy was third- and fourth-generation cephalosporins, followed by piperacillin/tazobactam. The modification of first-line antimicrobial therapy was performed in 56.9% of IEs. Granulocyte-colony stimulating factor was administered in 53.8% of IEs, and intravenous immunoglobulins were administered in 62.5% of IEs. One patient required admission to the intensive care unit. No infection-related mortality was reported. *Conclusions:* ALL patients have frequent IEs. Close monitoring, the identification of risk factors, the rapid empirical use of antibiotics in febrile neutropenia, and the timely modification of antimicrobial therapy play key roles in reducing infection-related morbidity and mortality in children with ALL.

## 1. Introduction

Acute lymphoblastic leukemia (ALL) is the most common malignant disease in children, accounting for 25% of all pediatric malignancies [1,2]. Contemporary treatment provides a cure in more than 90% of children with ALL [3]. Antileukemic therapy, however, has been associated with many side effects, most often myelosuppression and an increased risk of bacterial, viral, fungal, and parasitic infections. Therefore, the appropriate prevention and treatment of infections are key components of supportive therapy in pediatric oncology.

The aim of the study was to examine epidemiological, clinical, and microbiological characteristics of infections in children with ALL.

## 2. Materials and Methods

This retrospective study included 23 patients newly diagnosed with ALL (16 (69.6%) males and 7 (30.4%) females, aged 1.3 to 12.2 years) who were treated from 1 January 2015 to 31 December 2020 at the Division of Hematology, Oncology, and Clinical Genetics, Department of Pediatrics, Clinical Hospital Center (CHC) Rijeka, Croatia, and admitted for infectious complications. Patients with relapsed disease were excluded from the study.

The data were obtained from electronic records (Integrated Hospital Information System, IBIS) and the archives of CHC Rijeka. The following data were collected: demographic data (age and gender), degree and length of neutropenia, characteristics of infection (localization, clinical findings, number of febrile episodes, phase of chemotherapy protocol), microbiological isolates, type and length of antimicrobial therapy, length of hospitalization, and outcome of the treatment/disease. All infectious episodes (IEs) were accompanied by fever >38 °C, and all required hospitalization.

### 2.1. Ethical Statement

The study followed the guidelines of the Declaration of Helsinki and was approved by the Ethics Committee of CHC Rijeka (No. 2170-29-02/1-22-2, 27 June 2022).

### 2.2. Statistical Analysis

Microsoft Excel 2019 (v16.0) was used to collect and process data. Nominal and ordinal measurements are presented as frequencies (n) and proportions (%) and numerical measurements as average values and standard deviations.

## 3. Results

The mean age of the patients at diagnosis was 5.9 ± 2.8 years, of whom 20 children (87%) were less than 10 years of age. All patients were treated according to the ALL IC-BFM 2009 protocol. Twenty patients (87%) were stratified to the intermediate-risk (IR) group, 3 (13%) to the high-risk (HR) group, and none to the standard-risk (SR) group. All patients had implanted central venous catheters (CVCs): 18 patients (17.4%) with Port-a-Cath^®^, 4 patients (78.3%) with Broviac^®^, and 1 patient (4.3%) had both CVC types implanted (Table 1). All patients received prophylaxis with trimethoprim-sulfamethoxazole (TMP-SMX) for *Pneumocystis jiroveci* pneumonia.

There were 104 IEs reported on average, with 4.5 ± 2.3 IEs per patient. The largest number of patients (19 or 82.6%) had three or more IEs, three patients (13.1%) had two IEs, and one patient (4.3%) had one IE. In 48 IEs (46.2%), the absolute neutrophil count (ANC) was <500, and in 27 out of 48 IEs (25.9%), ANC was <200. In 28 IEs (58.3%), the duration of neutropenia was more than 7 days. Febrile neutropenia (defined as a body temperature ≥38 °C with an ANC of less than 500 cells/μL) was present in 43 IEs (41.4%) (Table 2). The causative agents were isolated in 67 IEs (64.4%), while in 80 IEs (76.9%), infection was only clinically documented. The onset of most IEs (62 or 59.6%) was during the hospital stay, and for the remaining 42 IEs (40.4%), hospitalization was indicated through emergency admission. The average number of IE-related hospitalizations was 3.9 ± 1.7 in IR patients and 8.3 ± 1.7 in HR patients. The highest occurrence of IEs was observed in the Early intensification phase (23.1%), followed by the Reinduction (21.2%), Maintenance (21.2%), Induction (16.3%), Consolidation (15.4%), and post-treatment periods (2.9%) (Table 2).

The two most common symptoms were cough (in 35 or 33.7% of IEs) and poor appetite (34 or 32.7% of IEs). Chills were reported in 22 IEs (21.2%), headaches in 21 IEs (20.2%), and nasal secretion in 20 IEs (19.2%). Other symptoms were diarrhea (in 17 or 16.3% of IEs), fatigue (15 or 14.4%), abdominal pain (14 or 13.5%), sore throat (10 or 9.6%), vomiting (9 or 8.7%), and sore ear (2 or 1.9%). The most frequent site of infection was the respiratory system (48.1% of IEs), followed by the occurrence of infection without a identified focus (23.1%), gastrointestinal tract (8.7%), skin (7.7%), and urinary tract (5.8%). Bacteria were isolated in 49 IEs (47.1%), combined pathogens were documented in 10 IEs (9.6%), and the causative agent was not isolated in 37 IEs (35.6%) (Table 2). Viral and fungal agents were isolated in 3.9% of all IEs each. Blood cultures were performed in 82 IEs (78.8%) and were negative in 75 IEs (91.5%). In four out of seven positive blood cultures, coagulase-negative *Staphylococcus* sp was isolated (accounting for 57.1% of positive cultures). In the remaining three positive blood cultures, *Acinetobacter baumannii*, *Pseudomonas* sp., and group A *Streptococcus pyogenes* were isolated (21.4% each). A total of 49 respiratory tract specimens were collected by nasopharyngeal swabs, pharyngeal swabs, and sinus aspirates. In the collected samples, the most frequently isolated bacteria were *Staphylococcus* sp (in 25 or 51% of samples), followed by *Candida albicans* (5 samples or 10.2%) and *Corynebacterium* sp. in 4 samples (8.2%). *Streptococcus pneumoniae* was isolated in three samples (6.1%), and *Enterobacter cloacae*, *Streptococcus mitis*, *Moraxella catarrhalis,* and *Enterococcus faecalis* were isolated in two samples (4.1%) each. *Klebsiella oxytoca*, *Streptococcus constellatus*, *Pseudomonas aeruginosa*, and *Bacillus* sp. were isolated in one sample each (2%). Urine cultures were taken in a total of 86 IEs and were positive in 11 cases (12.8%). The most frequent pathogen was *Pseudomonas aeruginosa* (27.3%). *Escherichia coli*, *Proteus vulgaris*, *Enterococcus faecalis,* and ESBL-producing *Klebsiella pneumoniae* were isolated in eight cases. A total of nine samples were taken from the skin, of which four were negative (44.4%). The most frequent isolated skin pathogen was *Enterococcus faecium* (in 3 or 33.3% of cases), followed by *Escherichia coli* in 2 cases (22.2%). *Enterococcus faecalis* and *Staphylococcus aureus* were isolated in one sample each (11.1%). Stool cultures were taken in 40 IEs and were positive in 22 IEs (55%). *Clostridium difficile* was the most common isolated pathogen in feces (40.9%), followed by *Candida* sp. (27.3%), Rotavirus (18.2%), *Campylobacter jejuni* (9.1%), and Norovirus (4.6%).

Chest X-rays were performed in 33 IEs (31.7%), with pathological findings in 12.1% of cases [Table 2].

Antimicrobial therapy was administered in 102 IEs (98.1%). The average duration of treatment was 8.5 ± 4.8 days. In 53.8% of IEs, patients received monotherapy as the first-line treatment, with the two most common antibiotics being cefepime and ceftriaxone (59.8% of IEs). Piperacillin/tazobactam was administered in 12.8% of IEs, azithromycin in 7.8%, and meropenem in 5.9% of IEs. In the remaining 13.7% of IEs, the following antibiotics were used: cefpodoxime, ciprofloxacin, vancomycin, tobramycin, and amoxicillin. Antimicrobial therapy was modified in 44 IEs (43.1%) due to the persistence of fever and/or the subsequently obtained isolate. Antifungals were administered in 42 IEs (41.2%): in 7 IEs (16.7%) as therapy and in 35 IEs (83.3%) as prophylaxis. The most frequent antifungal agent was fluconazole (in 85.7% cases), followed by micafungin (7.1%), voriconazole (4.8%), and caspofungin (2.4%).

Granulocyte-colony stimulating factor (G-CSF) was administered in 46.2% of IEs. Intravenous immunoglobulins (IVIG) were administered in 37.5% of IEs with documented secondary hypogammaglobulinemia (Table 2).

The average length of hospitalization was 10.4 ± 7.3 days. Only one patient required admission to the intensive care unit (ICU) due to severe complications related to infection (1% of IEs). No fatal outcomes were reported.

## 4. Discussion

ALL is the most common pediatric malignancy, with the peak age at diagnosis between three and six years and a slight male predominance [4]. The mean age at diagnosis in our study was 5.9 ± 2.8 years, with a higher prevalence of males (2.3:1). Similar age and gender distributions have been reported in other studies [4,5,6].

Based on the biological and clinical characteristics of the disease, as well as the response to the initial chemotherapy, pediatric ALL is stratified into three risk groups: SR, IR, and HR. Most of our patients were classified in the IR group, which corresponds to with literature data [7]. CVC was implanted in all patients, and all children received *Pneumocystis jiroveci* pneumonia prophylaxis. Both CVC use and TMP-SMX prophylaxis are considered standard components of the supportive therapy of contemporary protocols [8].

Children with ALL are at risk of bacterial, viral, fungal, and parasitic infections [9]. IEs are among the most common causes of hospitalization and are the most prevalent cause of death, after relapse. The largest number of patients in our study (82.6%) had three or more IEs, and most of these patients were in the HR group (8.3 ± 1.7 IEs), which received more aggressive chemotherapy. A similar distribution of IEs was described by Khan et al., where three or more hospital admissions accounted for 82.6% of ALL patients [10]. In the majority of our patients (59.6%), IE onset was in the hospital, which does not justify our previous approach of the careful monitoring of oncology patients with myelosuppression in hospital settings, despite infection prevention measures during antileukemic treatment.

In most European countries, children with ALL are treated according to BFM protocols, which include the following phases: Induction, Early intensification, Consolidation, Reinduction, and Maintenance therapy. Treatment is adapted to the risk groups and lasts for a total of 24 months. In relation to the phase of chemotherapy, 78 IEs (75%) were observed during intensive treatment (first 4 phases), and 22 IEs (21.2%) were documented during Maintenance. Our results coincide with the study of Bakhshi et al., in which 166 out of 222 IEs (74.7%) were recorded during the intensive phases, while 56 (25.3%) were recorded in the Maintenance therapy [6].

Malignant disease by itself and cytotoxic therapy have suppressive effects on different components of specific and non-specific immunity. The most significant risk factor for infections in cancer patients is neutropenia. ANC <100/microliter and duration of neutropenia greater than 7 days significantly increases the risk of severe infections. More than 50% of patients with ANC <500 had an infection, and 20% of them developed bacteremia if ANC < 100 [11]. The severity and length of neutropenia correlated with the severity of the clinical picture. In this study, neutropenia with ANC <500 was present in 45.2% of IEs, and 58.3% of cases had a duration longer than 7 days. Our results are comparable to the study of Khan et al., in which 61.2% of patients had ANC <500 and 58.8% of patients had neutropenia >7 days [10]. We documented febrile neutropenia in 43 IEs (41.4%), which is consistent with observations in other studies [8,12].

The causative agent was isolated in 67 IEs (64.4%), most commonly bacterial with Gram-positive strains. Blood culture was positive in 8.5% of samples. The most common infection site was the respiratory tract. *Staphylococcus* spp. was isolated in 57.1% of blood cultures and 56.2% of respiratory samples. The most common Gram-negative bacteria were *Pseudomonas aeruginosa* (6%). The predominance of Gram-positive bacterial infections is consistent with the literature data [5,6,10,13].

Antimicrobial therapy was administered in 98.1% of IEs, with an average duration of 8.5 ± 4.7 days. In 54.9% of IEs, monotherapy was given as the first-line treatment, which, considering the clinical stability of patients, complies with guidelines [10,14]. A modification to the therapy was required in 43.1% of IEs due to persistent fever and/or positive cultures. Guidelines on antifungal therapy are not universally accepted, vary significantly between institutions, and are subject to revision [14,15,16,17]. We administered antifungal prophylaxis to patients who were at high risk for invasive fungal infections and in the case of persistent fever in most IEs (83.3%). The reported mortality associated with infections is 2.5 to 6% [5,6,18,19]. In our study, only one IE required admission to the ICU, and no fatal outcome was reported. These results are likely due to the rigorous institutional infection-prevention measures and the immediate introduction of empirical antimicrobial therapy in children with febrile neutropenia.

## 5. Conclusions

In recent decades, great progress has been made in the treatment of children with ALL. Despite excellent results, infections remain the most common and potentially life-threatening complications of the antileukemic treatment. The prevention of infections in pediatric patients, close monitoring with the identification of those at risk, the rapid empirical use of antibiotics in children with febrile neutropenia, and the timely modification of antimicrobial therapy play a key role in reducing infection-related morbidity and mortality.

## Figures and Tables

**Table 1:** Patient characteristics.

Gender	N (%) *
Female	7 (30.4%)
Male	16 (69.6%)
Age (years)	N (%) *
<10	20 (86.9%)
≥10	3 (13.1%)
Risk group	N (%)
IR *	20 (86.9%)
HR *	3 (13.1%)
Type of CVC *	N (%)
Port-a-Cath^®^	18 (78.3%)
Broviac^®^	4 (17.4%)
Both	1 (4.6%)

Abbreviations: * number (N); central venous catheter (CVC); intermediate risk (IR); high risk (HR).

**Table 2:** Characteristics of infection.

Number of IEs (per patient)	N (%) *
3 and more	19 (82.6%)
2	3 (13.1%)
1	1 (4.3%)
ANC (cells/microlitre) *	N (%) *
>500	56 (53.8%)
200–500	21 (20.2%)
<200	27 (26%)
Duration of neutropenia (days)	N (%) *
<7	20 (41.7%)
>7	28 (58.3%)
Antibiotic prophylaxis	
TMP-SMX *	104 (100%)
Onset of IE *	N (%) *
In-hospital	62 (59.6%)
Out-of-hospital	42 (40.4%)
Phase of chemotherapy	N (%) *
Induction	17 (16.3%)
Early intensification	24 (23.1%)
Consolidation	16 (15.4%)
Reinduction	22 (21.2%)
Maintenance therapy	22 (21.2%)
Post-chemotherapy	3 (2.9%)
Site of infection	N (%) *
Respiratory tract	50 (48.1%)
Urinary tract	6 (5.7%)
Gastrointestinal tract	9 (8.7%)
Skin	8 (7.7%)
Blood (bacteriemia)	7 (6.7%)
Not determined	24 (23.1%)
Causative agent	N (%) *
Bacteria	49 (47.1%)
*Gram negative*	13
Most common agent: *Pseudomonas* sp.	4
Most common site: Urinary tract	3
Most common symptom: Abdominal pain	3
Most common antimicrobial:	
piperacillin/tazobactam/cefepime	3/3
*Gram positive*	36
Most common agent: *Staphyloccocus* sp.	22
Most common site: Respiratory tract	25
Most common symptom: Cough	14
Most common antimicrobial: cefepime	16
Viruses	4 (3.8%)
Fungi	4 (38%)
Combined	10 (9.6%)
Not isolated	37 (35.6%)
Blood culture	N (%) *
Positive	7 (8.5%)
Negative	75 (91.5%)
Urine culture	N (%)
Positive	11 (12.8%)
Negative	75 (87.2%)
Stool culture	N (%) *
Positive	22 (55%)
Negative	18 (45%)
Chest X-ray	N (%)
Pathological finding	4 (12.1%)
Normal finding	29 (87.9%)
Antimicrobial therapy	N (%)
Used	102 (98.1%)
Not used	2 (1.9%)
Type of antimicrobial therapy	N (%) *
Monotherapy	55 (53.9%)
Combined therapy	47 (46.1%)
Antifungal therapy	N (%) *
Therapeutic use	7 (16.7%)
Prophylactic use	35 (83.3%)
Supportive therapy	N (%)
IVIG *	39 (37.5%)
G-CSF *	48 (46.2%)

Abbreviations: * Absolute neutrophil count (ANC), Infectious episode (IE), Intravenous immunoglobulins (IVIG); Granulocyte-colony stimulating factor (G-CSF); number (N); trimethoprim-sulfamethoxazole (TMP-SMX).

## Data Availability

Data are unavailable due to privacy.
